# Mechanical Stress Induces Ca^2+^-Dependent Signal Transduction in Erythroblasts and Modulates Erythropoiesis

**DOI:** 10.3390/ijms22020955

**Published:** 2021-01-19

**Authors:** Francesca Aglialoro, Asena Abay, Nurcan Yagci, Minke A. E. Rab, Lars Kaestner, Richard van Wijk, Marieke von Lindern, Emile van den Akker

**Affiliations:** 1 Department of Hematopoiesis, Sanquin Research and Landsteiner Laboratory, Amsterdam UMC, University of Amsterdam, 1012 WX Amsterdam, The Netherlands; f.aglialoro@sanquin.nl (F.A.); a.abay@sanquin.nl (A.A.); n.yagci@sanquin.nl (N.Y.); m.vonlindern@sanquin.nl (M.v.L.); 2 Dynamics of Fluids, Experimental Physics, Saarland University, 66123 Saarbrücken, Germany; lars_kaestner@me.com; 3 Department of Clinical Chemistry and Haematology, University Medical Center Utrecht, 3584 CX Utrecht, The Netherlands; M.A.E.Rab@umcutrecht.nl (M.A.E.R.); R.vanWijk@umcutrecht.nl (R.v.W.); 4 Theoretical Medicine and Biosciences, Medical Faculty, Saarland University, 66123 Homburg, Germany

**Keywords:** PIEZO1, calcium signal transduction, mechanical stress

## Abstract

Bioreactors are increasingly implemented for large scale cultures of various mammalian cells, which requires optimization of culture conditions. Such upscaling is also required to produce red blood cells (RBC) for transfusion and therapy purposes. However, the physiological suitability of RBC cultures to be transferred to stirred bioreactors is not well understood. PIEZO1 is the most abundantly expressed known mechanosensor on erythroid cells. It is a cation channel that translates mechanical forces directly into a physiological response. We investigated signaling cascades downstream of PIEZO1 activated upon transitioning stationary cultures to orbital shaking associated with mechanical stress, and compared the results to direct activation of PIEZO1 by the chemical agonist Yoda1. Erythroblasts subjected to orbital shaking displayed decreased proliferation, comparable to incubation in the presence of a low dose of Yoda1. Epo (Erythropoietin)-dependent STAT5 phosphorylation, and Calcineurin-dependent NFAT dephosphorylation was enhanced. Phosphorylation of ERK was also induced by both orbital shaking and Yoda1 treatment. Activation of these pathways was inhibited by intracellular Ca^2+^ chelation (BAPTA-AM) in the orbital shaker. Our results suggest that PIEZO1 is functional and could be activated by the mechanical forces in a bioreactor setup, and results in the induction of Ca^2+^-dependent signaling cascades regulating various aspects of erythropoiesis. With this study, we showed that Yoda1 treatment and mechanical stress induced via orbital shaking results in comparable activation of some Ca^2+^-dependent pathways, exhibiting that there are direct physiological outcomes of mechanical stress on erythroblasts.

## 1. Introduction

Transfusion of donor-derived red blood cells (RBC) is the oldest and most frequent form of cell therapy. However, there is an unmet demand for cultured red blood cells (cRBC) for transfusion purposes, especially to increase the availability of erythrocytes carrying rare blood group antigens for alloimmunized patients [1]. Additionally, cRBC can be loaded or genetically altered to contain or express therapeutic molecules for specialized delivery [2,3,4]. These applications will require large scale expansion and differentiation of erythroblasts in bioreactors. The fundamental advantage of stirred bioreactors is a controlled perfusion of nutrients and gas overcoming the limits set by surface dependency and diffusion rates in conventional cultures. Although several protocols for in vitro erythroid culture have been established, it is important to emphasize that these protocols may not be directly translatable to turbulent bioreactor setups [5,6,7,8]. One major difference between the currently used stationary suspension cultures and dynamic cultures in stirred bioreactor setups is the application of mechanical stress due to turbulent fluid flow, cell-cell, and cell wall collisions. Therefore, the understanding of cellular processes downstream of mechanosensitive channels is crucial in transferring erythroblast cultures to bioreactors.

PIEZO1 (or FAM83A) is a homo-trimeric, mechanosensitive, non-selective cation channel conserved among species. Orthologues are present in eukaryotic and prokaryotic organisms but not in yeast [9,10,11]. Its expression profile is prominent in stretch or movement-related tissues such as lung or bladder but PIEZO1 is also functionally expressed in low copy numbers on erythrocytes [9,12]. Activation of PIEZO1 of erythrocytes causes an influx of cations. As a result, Ca^2+^ activates the Gardos channel (KCNN4, KCa3.1). This results in loss of intracellular K^+^ and subsequently of water, leading to dehydration of erythrocytes [13]. The reduced erythrocyte volume may facilitate cell passage through narrow capillaries [14]. The ability to restore the cell volume is crucial for cell viability [15]. Recently, we have shown that PIEZO1 is functional in erythroblasts, and plays a role in inside-out integrin activation [16]. Gain-of-function mutations within *PIEZO1* cause prolonged opening of the PIEZO1 channel upon activation, and are associated with an autosomal dominant disease called Hereditary Xerocytosis (HX) [12,13,17]. HX patients suffer from a variety of symptoms including hemolytic anemia. Different mutations are reported for *PIEZO1*, including c.6262C>G p.(Arg2088Gly), c.7367G>A p.(Arg2456His), and c.1792G>A p.(Val598Met) [18,19,20,21,22]. Although the mutations are located in different PIEZO1 regions, they all prolong the Ca^2+^ flux due to slower channel inactivation [23]. 

PIEZO1 activation during hematopoiesis reduces erythroid specification [9,24]. This raises the question of whether PIEZO1 has a function in erythroblasts and whether dysfunction or hyperactivation of this channel affects erythropoiesis. Ca^2+^ signaling is essential for erythropoiesis [25,26,27,28]. Ca^2+^ influx regulates the number of CFU-E/BFU-e, and is a necessary step in the commitment to terminal differentiation and in the enucleation process [29,30]. Ca^2+^ influx can mediate a multitude of signaling events including activation of calmodulin and Ca^2+^-dependent PKCs (Protein Kinase C). For instance, the calcineurin-nuclear factor of activated T cells (NFAT) pathway decreases KLF1 expression, thereby inhibiting erythropoiesis [31]. In contrast, activation of Ca^2+^-dependent PKCs positively regulates responsiveness to EPO and can activate survival proteins like STAT5 [32,33,34,35].

The only known physiological agonist of PIEZO1 is mechanical activation by shear stress. Recently, a screen of 3.25 million compounds identified the PIEZO1 agonist Yoda1 that binds to PIEZO1 but not its homologue PIEZO2 [36]. Yoda1 increases Ca^2+^ influx through PIEZO1 by stabilizing its open conformation [10]. Having confirmed PIEZO1 expression at the RNA level, we investigated the role of PIEZO1 during turbulent, orbitally shaken erythroid cultures [8]. PIEZO1 activation increased intracellular Ca^2+^ and led to activation of NFATc2 and positive modulation of EpoR signaling pathways (STAT5, MAPK), which decreased erythroblast survival and proliferation. The same signaling pathways were activated in stirred bioreactors, which needs to be taken into consideration concerning efforts for large scale production of red blood cells in turbulent bioreactor settings. 

## 2. Results

### 2.1. Extended PIEZO1 Activation by Yoda1 and Mechanical Stress Negatively Affects Erythroblasts Proliferation

Erythroblasts were cultured from peripheral blood mononuclear cells (PBMC) in the presence of erythropoietin (Epo), stem cell factor (SCF) and dexamethasone (dex), conditions that inhibit terminal differentiation and maintain erythroblasts in an immature stage expressing CD49d and CD235a (Supplementary Figure S1A, protocol from Heshusius et al. 2019) [8]. These cells express PIEZO1, as detected by flow cytometry and Western blot (Supplementary Figure S1B,C). Neither the HX mutations nor the activation of PIEZO1 by Yoda1 changed PIEZO1 expression levels (Supplementary Figure S1C). Activation of PIEZO1 by Yoda1 increased intracellular Ca^2+^ levels in CD71^+^CD235^dim^ erythroblasts, which was enhanced by PIEZO1 mutations in HX patient-derived erythroblast (Supplementary Figure S2A,B).

As PIEZO1 is the most abundantly expressed known mechanosensor on erythroblasts, it is the prime candidate to investigate how shear stress affects cellular processes. We compared activation of PIEZO1 by its agonist Yoda1 to mechanical stress. Then, erythroblasts were cultured for 4 days in flasks placed on an orbital shaker transferring an estimated maximum wall shear stress of ~1.8 Pa (Supplementary Figure S3). This estimation is comparable to maximum shear values measured in bioreactors that are commonly used in mammalian cell cultures (see Discussion: How to quantify mechanical stress?).

**Figure 1:**
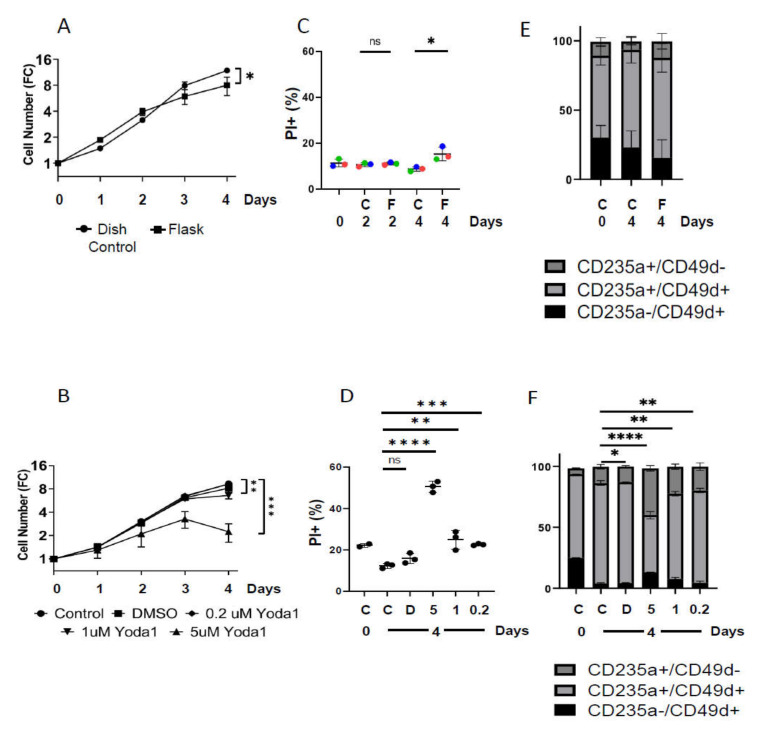
Mechanically stimulated and Yoda1-treated erythroblasts proliferation is impaired. Erythroblasts were cultured in flasks on the orbital shaker at 300 RPM (Flask: F) and in dishes in static control condition (Dish Control: C) for 4 days (**A**,**C**,**E**). In parallel, erythroblasts were cultured in absence (Control: C) or the presence of 0.2 μM (0.2), 1 μM (1) and 5 μM (5) Yoda1 or the solvent DMSO (DMSO: D) (**A**,**B**,**D**,**F**) Cell counts were assessed daily, cell number was maintained below 1,5 × 106 by daily dilution and cumulative cells were calculated (depicted as fold change (FC)). (**C**,**D**) Cell death was assessed by Propidium Iodide (PI) staining at day 0, 2, or 4 for orbital shaking, and at day 0 and 4 for Yoda1 treatment. Significance refers to the most mature CD235a^+^/CD49d- population. (**E**,**F**) Erythroblasts were stained with Pacific Blue-labeled anti CD49d and PE-labeled anti CD235a (GPA) antibodies and analyzed on the flow cytometer. Expression is plotted as percentage of more immature (black; CD235a^-^/CD49d^+^), and more mature erythroblasts (light grey; CD235a^+^/CD49d^+^ and dark grey; CD235a^+^/CD49d^-^). All experiments were performed in triplicates, error bars indicate standard deviation. For orbital shaker experiments paired t-tests, and for Yoda1 experiments unpaired *t*-tests were performed to assay significance between conditions (* *p* < 0.05; ** *p* < 0.01; *** *p* < 0.001; **** *p* < 0.0001).

Prolonged activation of PIEZO1 by Yoda1 led to reduced proliferation compared to control cells in a dose-dependent manner, whereas orbital shaking reduced cell proliferation in a comparable way to 1 µM Yoda (Figure 1A,B). Cells cultured on the orbital shaker displayed a 30% reduction of proliferation compared to controls at the end of 4 days of expansion, which was similar to Yoda1-induced (1 µM) reduced proliferation. The viability of cells in the orbital shaker and cells treated with 1 µM Yoda1 was slightly lower than controls. The percentage of PI^+^ cells, a measurement of cell death, was increased by 7 ± 2% after 4 days of shear stress, and 13 ± 3% after 4 days exposure to 1 µM Yoda1 (Figure S1C,D). The percentage of mature CD49d^−^/CD235a^+^ population was not significantly altered in response to shear stress but was significantly increased by Yoda1 incubation in a dose-dependent manner (Figure 1E,F).

A biologically significant decrease in cell proliferation accompanied by an increase in cell death increased the percentage of more mature CD49d^−^/CD235a^+^ erythroid cells, which was only observed at 5 µM Yoda (>50% PI+, Figure 1B,D,F).

### 2.2. Mechanical Stimulation and Yoda1 Incubation Induce Ca^2+^-Dependent Signaling Pathways

**Figure 2:**
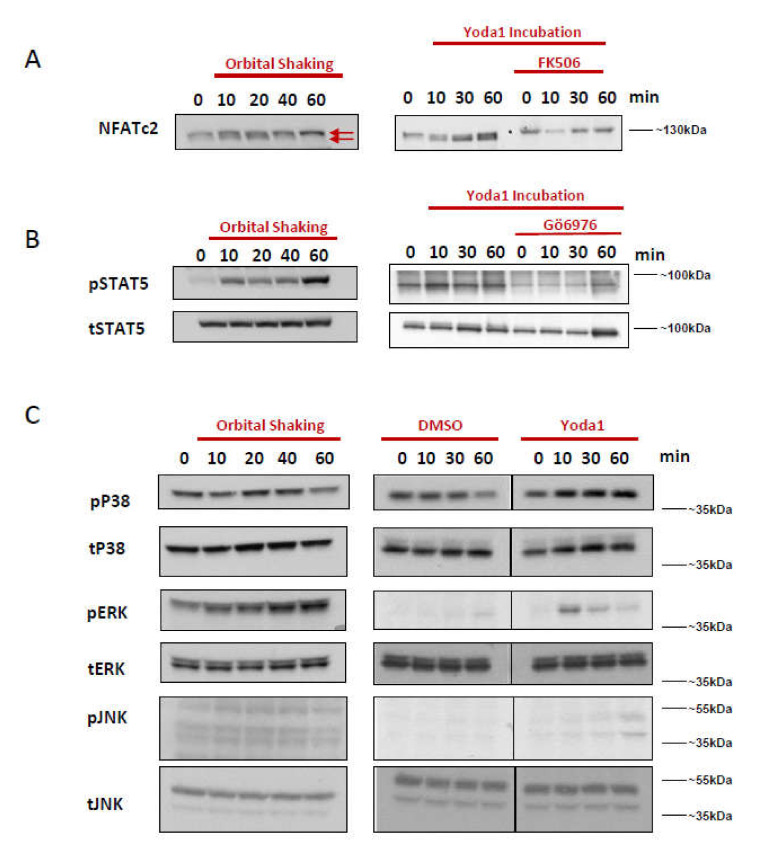
Mechanical stimulation and Yoda1 incubation induces Ca^2+^-dependent signaling pathways. Erythroblasts were either cultured on an orbital shaker set at 300 RPM and sampled at 0, 10, 20, 40, and 60 min, or stimulated with 1 μM Yoda1 with or without specific inhibitors and sampled at 0, 10, 30, 60 min. Total protein lysates were subjected to SDS-polyacrylamide gel electrophoresis and western blotting. (**A**) Some of the erythroblasts were pretreated with 1 μM FK506 before Yoda1 incubation. Blots were stained with antibodies against NFATc2 (arrows indicate the slower migrating phosphorylated, and faster migrating unphosphorylated isoforms). (**B**) Some of the erythroblasts were pretreated with 500 nM Gö6976 before Yoda1 incubation. Blots were stained with antibodies against tyrosine-phosphorylated-STAT5 (pSTAT5) and total STAT5 (t-STAT5). (**C**) Blots were stained with antibodies against phosphorylated JNK (Thr183/Tyr185) and total JNK, phosphorylated ERK1/2 (Thr202/Tyr204) and total ERK1/2, phosphorylated P38 (Thr180/Tyr182) and total p38. With orbital shaking even after a long exposure, nothing other than background bands were visible for phosphorylated JNK

Activation of PIEZO1 increased Ca2+ influx (Supplementary Figure S2A). Next we investigated whether signal transduction induced by orbital shaking of erythroblasts is comparable to Ca2+-dependent pathways induced in erythroblasts by the PIEZO1 agonist Yoda1. Important effectors of Ca^2+^ signaling are the NFAT transcriptional regulators [31,37]. Calmodulin activates the phosphatase calcineurin, which dephosphorylates critical residues in NFAT leading to NFAT nuclear localization and induction of target genes. NFATC2 dephosphorylation can be observed as a band with lower molecular weight [38]. Orbital shaking induced dephosphorylation of NFATC2 in 10 min, which was followed by phosphorylation back to basal levels within 60 min. Incubation with Yoda1 (1 µM) also induced NFATC2 dephosphorylation within 10 min. However, the dephosphorylated state was maintained for at least 60 min. Pre-incubation with the calcineurin inhibitor FK506 (Tacrolimus) resulted in a block of NFATC2 dephosphorylation confirming that the calcineurin pathway is downstream of PIEZO1 channel activity (Figure 2A). Erythroblasts of HX patients (TAP7; c.7367G>A, p.Arg2456His) also displayed increased NFATC2 dephosphorylation, which could be further increased upon treatment with Yoda1 (Supplementary Figure S4). 

Epo-induced signal transduction in erythroblasts can be modulated by the activation status of calcium-dependent PKCs [32]. Orbital shaking increased steady state phosphorylation of STAT5, which was maintained for at least 60 min (Figure 2B). Similarly, Yoda1 transiently increased STAT5 phosphorylation. Pretreatment of erythroblast cultures with an inhibitor of Ca^2+^-sensitive PKCs (Gö6976) blocked STAT5 phosphorylation thus linking Yoda1 induced Ca^2+^ influx to PKC-dependent modulation of STAT5, possibly through regulation of EPO-responsiveness (Figure 2B).

MAPK pathways such as P38, ERK1/2, and JNK are Epo-receptor downstream events, but they can also be directly downstream of Ca^2+^-sensitive PKC [35,39,40,41]. Yoda1 incubation weakly induced phosphorylation of p38 for at least 60 min, which was less clear for orbital shaking (Figure 2A). Phosphorylation of ERK was more prominent. Orbital shaking induced ERK phosphorylation for 60 min, whereas Yoda1 incubation induced transient ERK phosphorylation with a peak at 10 min. JNK was not phosphorylated by orbital shaking, whereas Yoda1 incubation induced JNK phosphorylation reaching a maximum at 60 min (Figure 2C). Yoda1-induced phosphorylation of JNK could be inhibited with pretreatment of 500 nM of Gö6976 (Supplementary Figure S5). MAPK pathways were also studied in erythroblasts from HX patients with or without Yoda1 incubation. The results displayed a very similar activation profile to healthy erythroblasts. P38 phosphorylation followed the same kinetics upon Yoda1 incubation of control and patient samples. Compared to the healthy control, HX-derived erythroblasts showed increased JNK and ERK phosphorylation at steady state, which was further amplified by Yoda1 treatment. The dynamics of JNK and ERK phosphorylation over time were similar between healthy and HX erythroblasts (Supplementary Figure S4). Orbital shaking experiments were repeated with three donors, and the same phosphorylation patterns were observed (Supplementary Figure S6). Overall, the data indicate that pharmacological PIEZO1 activation and orbital shaking elicit similar downstream signaling except for JNK activation.

### 2.3. Intracellular Ca^2+^ Chelation Prevents Shear-Induced Signaling Pathways

**Figure 3:**
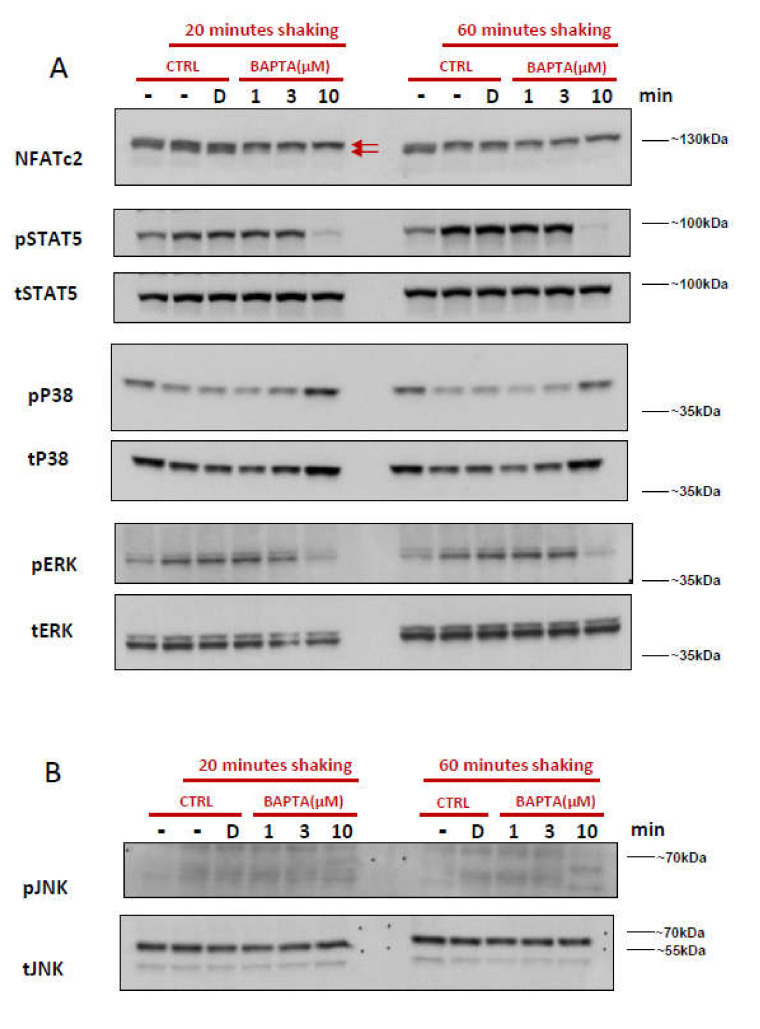
Intracellular Ca^2+^ chelation prevents shear-induced signaling pathways. Cultured erythroblasts were preincubated for 10 min under static conditions, and subsequently orbitally shaken at 300 rpm for 20 or 60 min (first lane = Time 0). These cultures were left untreated (CTRL, -), treated with solvent DMSO (CTRL, D), or pre-incubated with 1, 3, and 10 μM of the Ca^2+^ chelator BAPTA-AM (BAPTA(μM), 1, 3, 10). Total protein lysates were subjected to SDS-polyacrylamide gel electrophoresis and western blotting. (**A**) Blots were stained with anti-NFATc2, anti-p-STAT5 and total-STAT5, anti-p-ERK1/2 (Thr202/Tyr204), anti-Total-ERK1/2, anti-p-P38 (Thr180/Tyr182), anti-total p38. (**B**) Blots were stained with anti-pJNK (Thr183/Tyr185) and anti-Total-JNK.

PIEZO1 conducts Ca^2+^ but also can conduct other cations like Mg^2+^ [42,43]. To confirm the specificity of mechanical stress-induced signal transduction to influx of Ca^2+^, we employed an intracellular Ca^2+^ chelator. CD71^+^CD235^dim^ erythroblasts were pre-incubated with increasing concentrations of a highly selective intracellular Ca^2+^ chelator, BAPTA-AM. High concentration of BAPTA-AM (100 µM; 1 h) resulted in an average of 34% PI+ cells overall signifying a lower viability, and specifically decreased the number of CD71+/CD235+ cells by 30–40%. Chelation with 10 μM BAPTA-AM did not affect viability, and the CD71/CD235 profile was similar to control cells (Supplementary Figure S7A,B). Dephosphorylation of NFAT and phosphorylation of ERK and STAT5 induced by orbital shaking was abrogated by pretreatment of the erythroblasts with 10 µM BAPTA-AM (Figure 3A). Lower BAPTA-AM concentrations prevented the mechanically induced dephosphorylation of NFATc2 in a dose-dependent manner. In contrast, phosphorylated p38 was upregulated with 10 μM BAPTA-AM incubation, whereas phosphorylated p38 is downregulated in control samples (Figure 3A). Similarly to what we observed in previous experiments, we did not detect phosphorylation of JNK following 20 or 60 min of shaking. However, treatment of erythroblasts with the higher concentration of 10 μM BAPTA-AM followed by 60 min of shaking resulted in phosphorylation of JNK (Figure 3B). The data confirm that, specifically, Ca^2+^ influx is the initiating event that triggers activation of ERK, NFATC2, and STAT5 in the setup of an orbital shaker.

## 3. Discussion

Large scale production of cRBC for transfusion and therapy purposes requires the use of bioreactors, which exposes erythroblast to fluidic stress from, for instance, impellers and capillary flow. The major known sensor of mechanical stress in erythropoiesis is PIEZO1. Here, we confirmed that in vitro cultured erythroblasts express the mechanosensitive channel PIEZO1 and that activation results in Ca^2+^-dependent downstream signaling [8]. We show that mechanical stress reduced proliferation and increased differentiation of committed erythroblasts to an extent that is similar to the activation of PIEZO1 by 1 µM Yoda1. Transition from static cultures to turbulent orbital shaking induced Ca^2+^-dependent signaling cascades such as the Calcineurin/NFAT and STAT5 pathways, similar to the exposure of erythroblasts to the pharmacological PIEZO1 agonist Yoda1 (Figure 2) [24]. This indicates that fluid turbulence, as experienced in bioreactors or orbital shaker setups, leads to a specific response in erythroblasts, which is important to consider upon upscaling and optimizing erythroid cultures to non-stationary platforms. In addition, we provide specific molecular markers to determine the mechanosensing-induced signal transduction, providing a model system to study the physiology of erythroblasts upon repeated mechanical stimulation.

### 3.1. How to Quantify Mechanical Stress?

Due to the chaotic nature of turbulent flows, fluidic forces in a bioreactor are highly variant. Fluid flow can be computationally simulated/modelled, estimated to an average value, or directly measured (to be averaged) via particle image velocimetry (PIV). For the setup of stirred-tank bioreactors, mechanical stress will greatly vary depending on the position within the reactor. Near the impeller region, the fluid flow will be much faster than near the wall region. For the approximations in this study we used the maximum estimates. From COMSOL simulations with rotating domain approach and k-ε turbulent model, we estimated an average maximum shear stress of 1.04 Pa near the high stress impeller region in one of the bioreactors we currently employ (AppliFlex ST bioreactors, Applikon Biotechnology BV, Delft, The Netherlands). Odeleye et al. measured values up to 2.5 Pa in Mobius^®^ 3 L CellReady (Merck Millipore, Burlington, MA, USA) commercial bioreactor via PIV technique [44]. For this paper, we decided to use a setup that yields 1.84 Pa of maximum wall shear stress, which is a comparable value to both our computational simulation and an experimental measurement performed in a commercial bioreactor that is commonly used for mammalian cells. 

### 3.2. Control of Intracellular Ca^2+^ Levels

The delicate balance of intracellular Ca^2+^ is maintained by Ca^2+^ transporters in the plasma membrane as well as by transporters in Ca^2+^ storing organelles, e.g., ER and mitochondria [45,46]. High cytoplasmic Ca^2+^ has been described to cause mitochondrial Ca^2+^ overload and release of caspase cofactors to induce apoptosis, which may have caused the observed cell death at high concentrations (5 μM) of Yoda1 treatment [47]. In contrast, very high concentrations of the intracellular Ca^2+^ chelator BAPTA-AM (100 μM) also induced cell death, indicating that erythroid cells are also sensitive to large drops in free cytoplasmic Ca^2+^. The results confirm that the intracellular Ca^2+^ levels are tightly balanced during erythropoiesis and inappropriate deviations from the normal physiological signaling lead to loss of cell viability, differentiation and/or proliferation defects. We observed that application of mechanical stress using orbital shaking and treatment with 1 μM Yoda1 resulted in similar effects in signaling, most of which could be manipulated by intracellular chelation of Ca^2+^ (Figure 3). Thus it was of interest to compare the intracellular Ca^2+^ levels between these two conditions. There is a good amount of evidence to suggest that Yoda1 induced opening of PIEZO1 is stable [48,49]. So relative intracellular Ca^2+^ quantification in erythroblasts in presence of Yoda1 using a Ca^2+^ bound dye (e.g., FLUO-4) is possible (Supplementary Figure S2). However, this measurement technique is not directly applicable for cells from orbitally shaken setup because the mechanically induced Ca^2+^ uptake can be quickly dissipated as the intracellular Ca^2+^ is very tightly and rapidly regulated. Removing the cells from the mechanical stress condition for sampling and staining can result in an almost immediate loss of mechanically increased Ca^2+^ concentration. The rapidity of such process was exemplified in erythrocytes passing through a small constriction, in which the constriction-induced Ca^2+^ uptake was quickly dissipated to nominal values upon relaxation [14]. We hypothesize that a similar rapid drop in cytoplasmic Ca^2+^ occurs when the sample is removed from the mechanical stress condition.

### 3.3. Signal Transduction Downstream of Mechanosensing

It is essential to understand cellular responses to mechanical stress in order to develop a bioreactor in which erythroid expansion and differentiation is optimized to yield sufficient RBC for downstream applications. Both mechanical stimulation and Yoda1 treatment increased phosphorylation of effector molecules dowmstream of the EpoR among which STAT5 and the MAPKinases -ERK and p38. Phosphorylation was blocked by the calcium-dependent PKC inhibitor Gö6976 and by the intracellular calcium chelator BAPTA (Figure 2B and Figure 3A) These data link mechanical sensing in turbulent fluidics settings to previously established connections between Ca^2+^ influx, PKC activation, and Epo-receptor signaling and suggests that the Ca^2+^ cascade triggered by either orbital shaking or Yoda1 regulates Epo-responsiveness [32]. This is in agreement with the notion that Ca^2+^ Ionophores increase the late CFU-E formation, which is inhibited by PKC inhibition [50]. STAT5 and ERK (MAPK routes) are central pathways in EpoR signaling, and regulate transcription of multiple target genes (e.g., BcL-xL) that are essential for erythropoiesis, particularly during terminal differentiation [35,39,51,52]. The increased phosphorylation of STAT5 may contribute to increased differentiation observed by Yoda1 and mechanical stress. Additionally, long term activation of PKCs, e.g., by orbital shaking, may promote RNA shuttling of specific transcripts (e.g., Elavl1) from the nucleus to the cytosol, thereby regulating erythropoiesis, which has been shown recently for GATA1, an essential transcription factor for erythropoiesis [53]. Although we uncover a clear link between PIEZO1-induced calcium influx and Epo-receptor signaling, it remains unknown how Ca^2+^-dependent PKCs control Epo-responsiveness. The immediate effects observed here on specific signaling pathways upon short term activation and inhibition probably rule out a role for transcription or translation in the modulation.

Besides these Epo-receptor downstream signal transduction pathways, also the calcium-dependent NFATc2 was activated upon PIEZO1 activation or orbital shaking. Of note, the expression of NFATc2 also seems to be increased, which cooperates with activation. NFATc2 is one of the members of the NFAT family that is directly activated by the Ca^2+^-dependent phosphatase calcineurin [54]. Dephosphorylation of NFATs leads to nuclear localization and target gene regulation. NFATc2 knockout mice were consistently found to be anemic and display increased splenic erythropoiesis suggesting a role for NFATc2 in terminal erythropoiesis [55]. 

NFATc1 is the predominantly expressed NFAT in murine erythroblasts, while NFATc2 expression is low (datamined from Kingsley et al. 2013) [56]. Interestingly, NFATc1 cooperates with STAT5 to induce KLF1 expression, an essential transcription factor during terminal erythroblast differentiation, indicating that STATs and NFATs may regulate specific target genes in a synergistic manner to positively regulate erythroblast differentiation [31,57]. The DNA binding motif of NFATc1 and NFATc2 is similar [58]. Interestingly, we did not observe NFATc1 dephosphorylation upon Yoda1 treatment of erythroblasts (Supplementary Figure S8). We hypothesize that the mechanical stress-induced and Ca^2+^-dependent NFATc2 activation and increased STAT5 phosphorylation may lead to upregulation of STAT5 and NFAT controlled target genes. The identity of these genes remains to be elucidated.

All mechanical stress-induced pathways could be efficiently blocked by the intracellular Ca^2+^ chelator BAPTA-AM, which proves a direct dependency on Ca^2+^. Employing BAPTA-AM was chosen due to the absence of a specific PIEZO1 channel inhibitor. Although GsMTx-4 is often used as an inhibitor of PIEZO1, it is not channel-specific. GsMTx-4 peptide incorporates itself into the lipid layer, and when the membrane is subjected to tension, it allows for partial relaxation by penetrating itself deeper into the membrane [43]. GsMTx-4 would partially inhibit all mechanosensitive channels and processes, besides changing membrane functionality.

**Figure 4:**
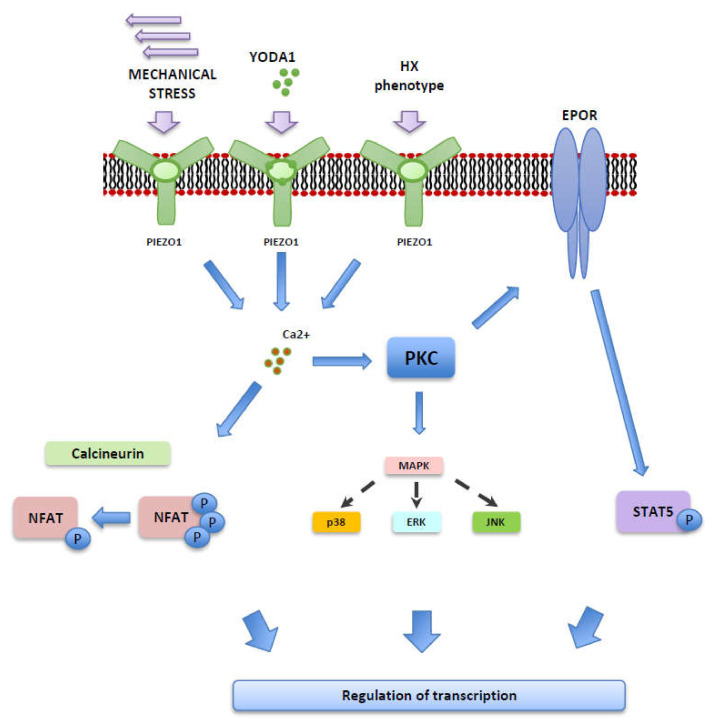
Overview of possible signal transduction downstream of PIEZO1 activation triggered by mechanical stress, upon the PIEZO1 agonist Yoda1 or the HX phenotype.

In conclusion, we demonstrated that mechanical stimulation and pharmacological activation of PIEZO1 on erythroblasts yield comparable results in terms of downstream signaling, which we show to be Ca^2+^-dependent (Figure 4). These pathways regulate many aspects of erythropoiesis as described above. These results are particularly important when designing bioreactor systems, where the cells will be subjected to repeated mechanical stress for long periods of time. Pathways that were activated by mechanical stimulation (STAT5, NFATC2, or MAPK routes) make excellent candidates to be used as biomarkers of mechanical stress in erythroblasts. Additionally, we propose that inhibition of the ERK pathway, which promotes cell death, could be implemented in non-stationary setups to counteract the lower cell viability. However, more research needs to be performed, especially concerning long term implications of inhibitor use. We hypothesize that similar signaling will be observed in any other turbulent fluid agitation methods used in bioreactors, like stirred-tank or gas-sparged. The biological consequences of these pathways should be monitored and potentially manipulated in bioreactors to increase efficiency.

## 4. Materials and Methods

### 4.1. Human Blood Sample

Peripheral Blood Mononuclear Cells (PBMCs) were purified by Percoll density separation, following the manufacturer’s protocol (GE Healthcare, Chicago, IL, USA). Informed consent was given in accordance with the Declaration of Helsinki, the Dutch National, and Sanquin Internal Ethic Boards. Blood from HX patients was collected as part of the RELEVANCE project (EC Grant Agreement 675117). The research on patient samples from University Medical Center (UMC) Utrecht was reviewed and approved by the Medical Ethical Review Board (MERB) from UMC Utrecht on 10 August 2017 (METC protocol 17/450). The HX patient had a mutation in PIEZO1 (c.7367G>A p.(Arg2456His)) and was splenectomized. Blood from healthy control donors was anonymously obtained using the approved medical ethical protocol of 07/125 Mini Donor Dienst, also approved by the MERB of UMCU. 

### 4.2. Erythroblast Cell Culture

Erythroblasts were cultured as previously described [Heshusius et al., 2019]. In short, isolated peripheral blood mononuclear cells (PBMCs) were cultured in the presence of EPO (2 U/mL; ProSpec, East Brunswick, NJ, USA), human recombinant Stem Cell Factor (100 ng/mL, supernatant SCF producing cell line), and dexamethasone (1 μM; Sigma, St. Louis, MO, USA) to pro-erythroblasts in Iscove’s Modified Dulbecco’s Medium with a reported Ca^2+^ concentration of 1.5 mM. This culture condition prevents differentiation and keeps the cells at the more immature CD49d^+^ phase. When indicated, cells were treated with different concentrations of Yoda1 (Sigma Aldrich, Munich, Germany), DMSO, or left untreated.

### 4.3. Orbital Shaking to Simulate Mechanical Stress

Orbital shaking was performed in 125 mL Corning Erlenmeyer cell culture flasks with ventilated caps (Sigma Aldrich, Munich, Germany) on an INB-101SRC Orbital Shaker (IKS International, Rosmalen, The Netherlands). For all experiments, 300 revolutions per minute (RPM) was used with 6.5 mL sample volume. The same volume was used for static dish controls in 6 cm dishes. When indicated, the intracellular Ca^2+^ chelator BAPTA-AM (Sigma Aldrich, Munich, Germany) was loaded into the erythroblasts. DMSO concentrations in culture never exceeded 0.1%.

### 4.4. Stimulation and Western Blot

Cells were counted with CASY (CASY^®^Model TTC, Schärfe System GmbH, Reutlingen, Germany), and treated with different Yoda1 concentrations (Sigma Aldrich, Munich, Germany), preceded or not by 15 min incubation with 500 nM Gö6976, or 1 μM FK506. For all western blot samples, cells were washed in ice-cold PBS and lysed in CARIN lysis buffer (20 mM Tris-HCl pH 8.0, 138 mM NaCl, 10 mM EDTA, 100 mM NaF, 1% Nonidet P-40, 10% glycerol). Lysates were boiled in Laemmli sample buffer and subjected to SDS-polyacrylamide electrophoresis, blotted using iBlot-PVDF blotting system (ThermoFisher Scientific, Bleiswijk, The Netherlands), and stained as indicated in the figure legends. 

### 4.5. Flow Cytometry and Ca^2+^ Measurement

Erythroblasts were resuspended in HEPES buffer (132 mM NaCl, 20 mM HEPES, 6 mM KCl, 1 mM MgSO_4_, 1.2 mMK_2_ HPO_4_) supplemented with 1% Bovine Serum Albumin (BSA) and incubated with primary antibodies for 30 min at 4 °C, and when needed followed by Goat-anti-Rabbit-PE secondary antibody (SantaCruz Biotechnology, Dallas, TX, USA). The following antibodies were used: antiCD71 (MiltenyiBiotec, Bergisch Gladbach, Germany), antiCD235a (OriGene Technologies, Rockville, MD, USA), antiCD49d (BD Biosciences, San Jose, CA, USA), PIEZO1 (Proteintech, Manchester, UK). Propidium Iodide was used as a life/dead marker (Thermo Fisher Scientific, Watlham, MA, USA). Measurements were done on FACS Canto II (BD Biosciences, Oxford, UK) and analyzed using FlowJo software (FlowJo v10; Tree Star, Inc., Ashland, OR, USA). For Ca^2+^ measurements, cells were loaded with 0.5 μM Fluo4-AM (ThermoFisher Scientific, Waltham, MA, USA) in HEPES buffer supplemented with 0.5% human serum albumin, 1 mMCaCl_2_, 1 mg/mL glucose for 30 min at 37 °C.

## Supplementary Materials

Supplementary materials can be found at https://www.mdpi.com/1422-0067/22/2/955/s1.
