# Supplementary material for: Mechanical Stress Induces Ca2+-Dependent Signal Transduction in Erythroblasts and Modulates Erythropoiesis
Source: Int J Mol Sci. 2021 Jan 19;22(2):955. doi: 10.3390/ijms22020955 (PMC7835781; doi:10.3390/ijms22020955)
Supplement: Supplementary file 1 [file ijms-22-00955-s001.pptx]

## Slide 1
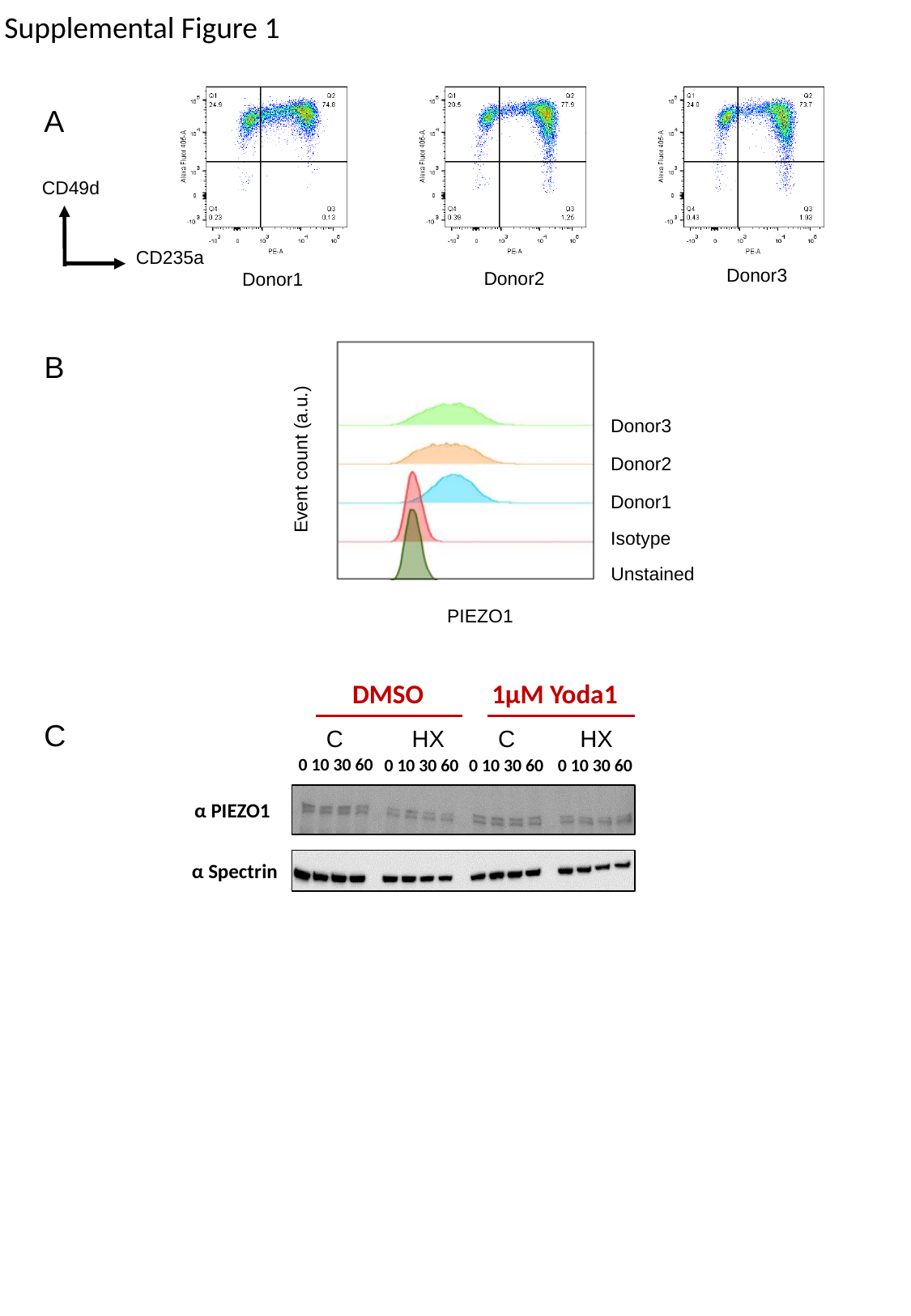

Supplemental Figure 1
A
CD49d
CD235a
Donor3
Donor2
Donor1
Event count (a.u.)
Isotype
Unstained
Donor3
Donor2
Donor1
PIEZO1
B
DMSO
1µM Yoda1
C
C
HX
C
HX
0 10 30 60
0 10 30 60
0 10 30 60
0 10 30 60
α PIEZO1
α Spectrin

## Slide 2
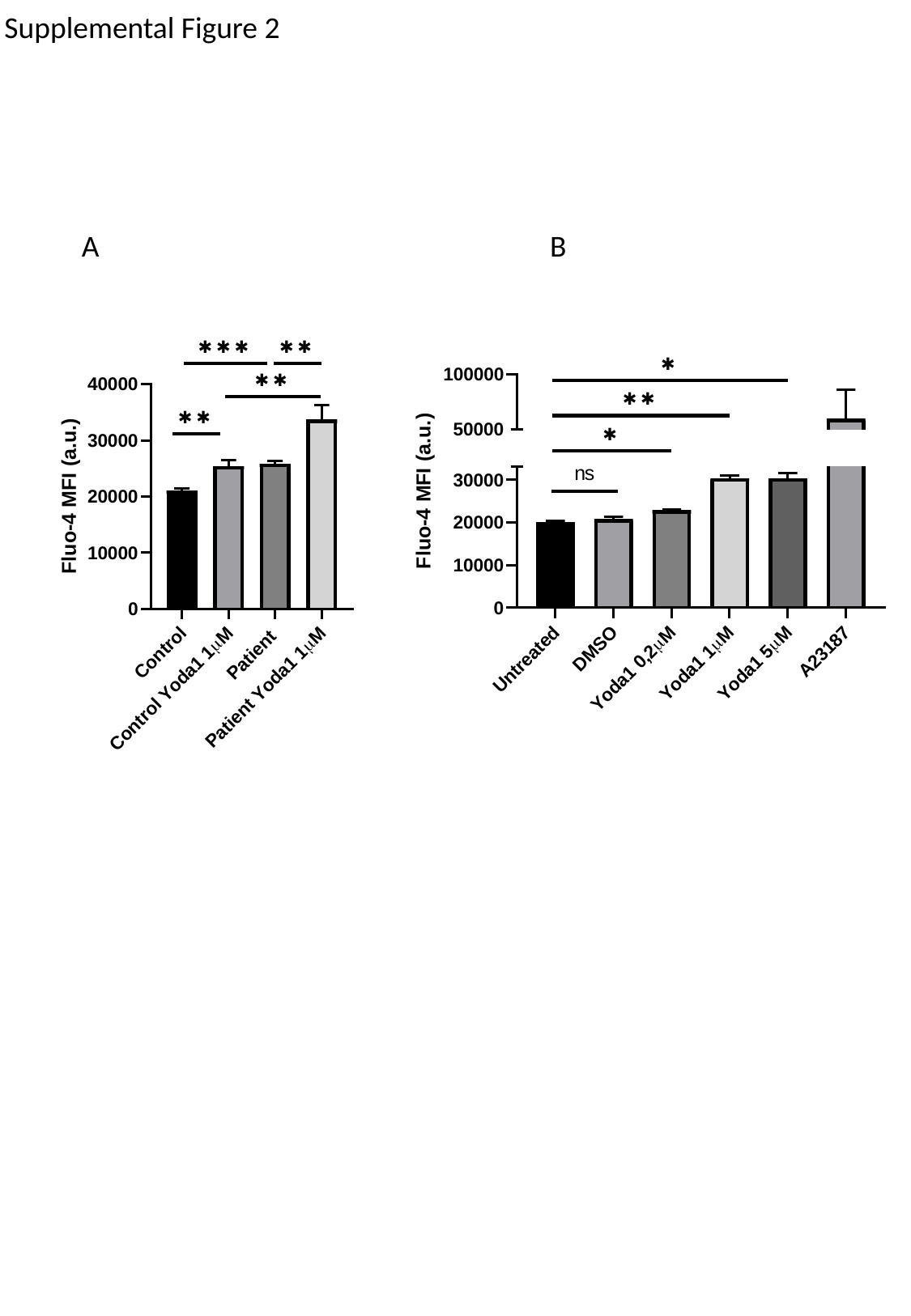

Supplemental Figure 2
A
B

## Slide 3
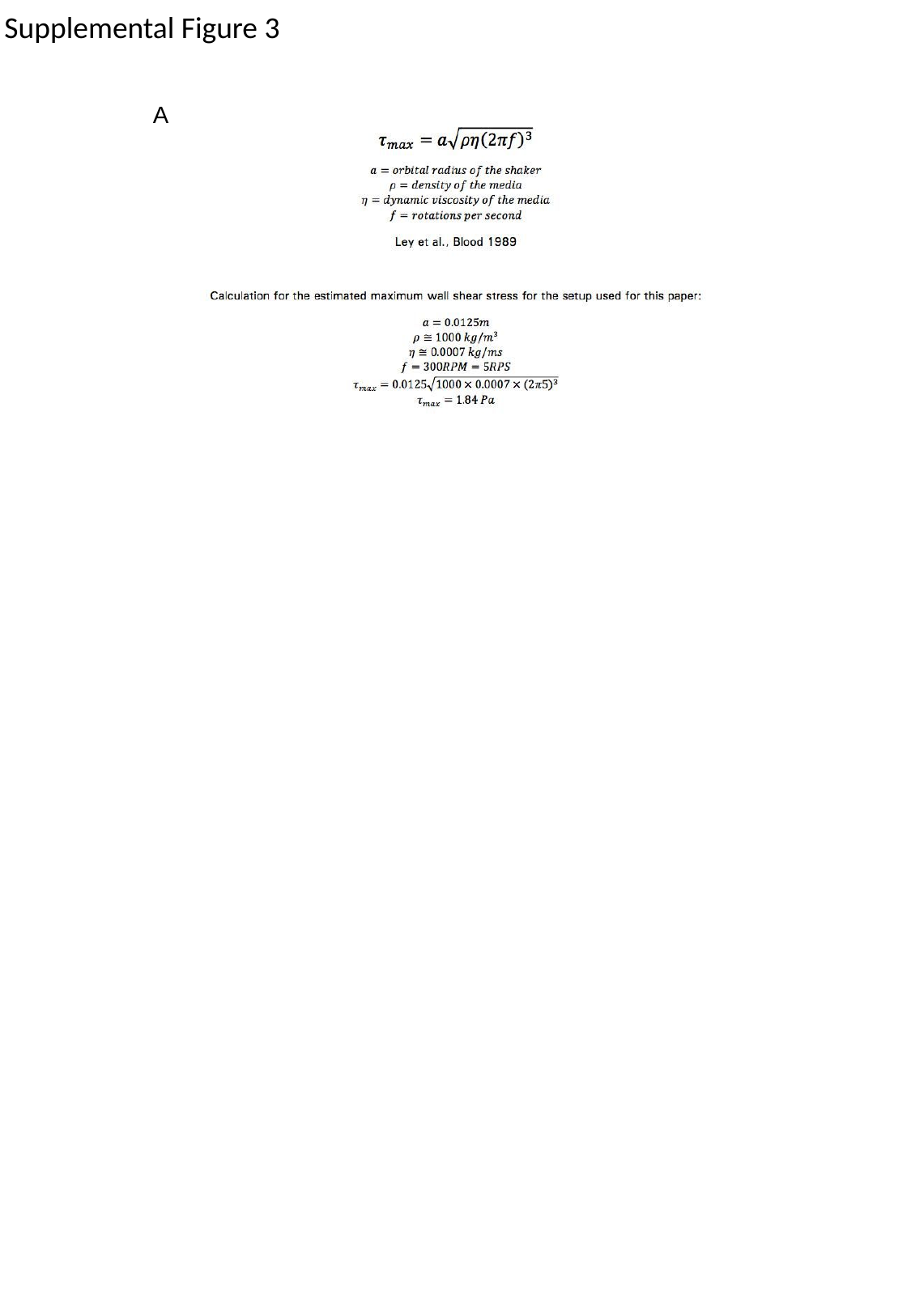

Supplemental Figure 3
A

## Slide 4
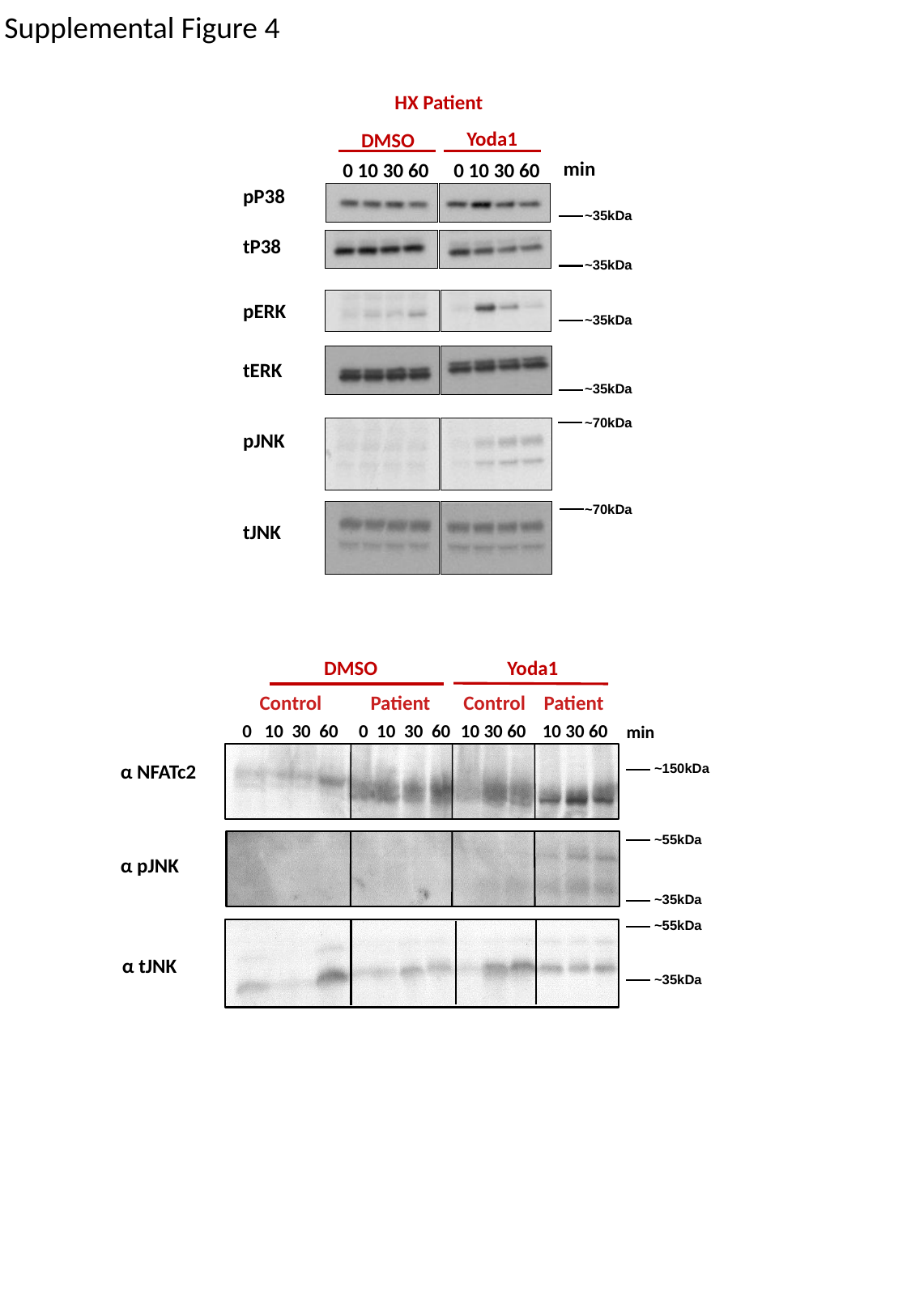

Supplemental Figure 4
HX Patient
Yoda1
DMSO
min
 0 10 30 60
 0 10 30 60
pP38
~35kDa
tP38
~35kDa
pERK
~35kDa
tERK
~35kDa
~70kDa
pJNK
~70kDa
tJNK
DMSO
Yoda1
Control
Patient
Control
Patient
 0 10 30 60
 0 10 30 60
 10 30 60
 10 30 60
min
α NFATc2
~150kDa
~55kDa
α pJNK
~35kDa
~55kDa
α tJNK
~35kDa

## Slide 5
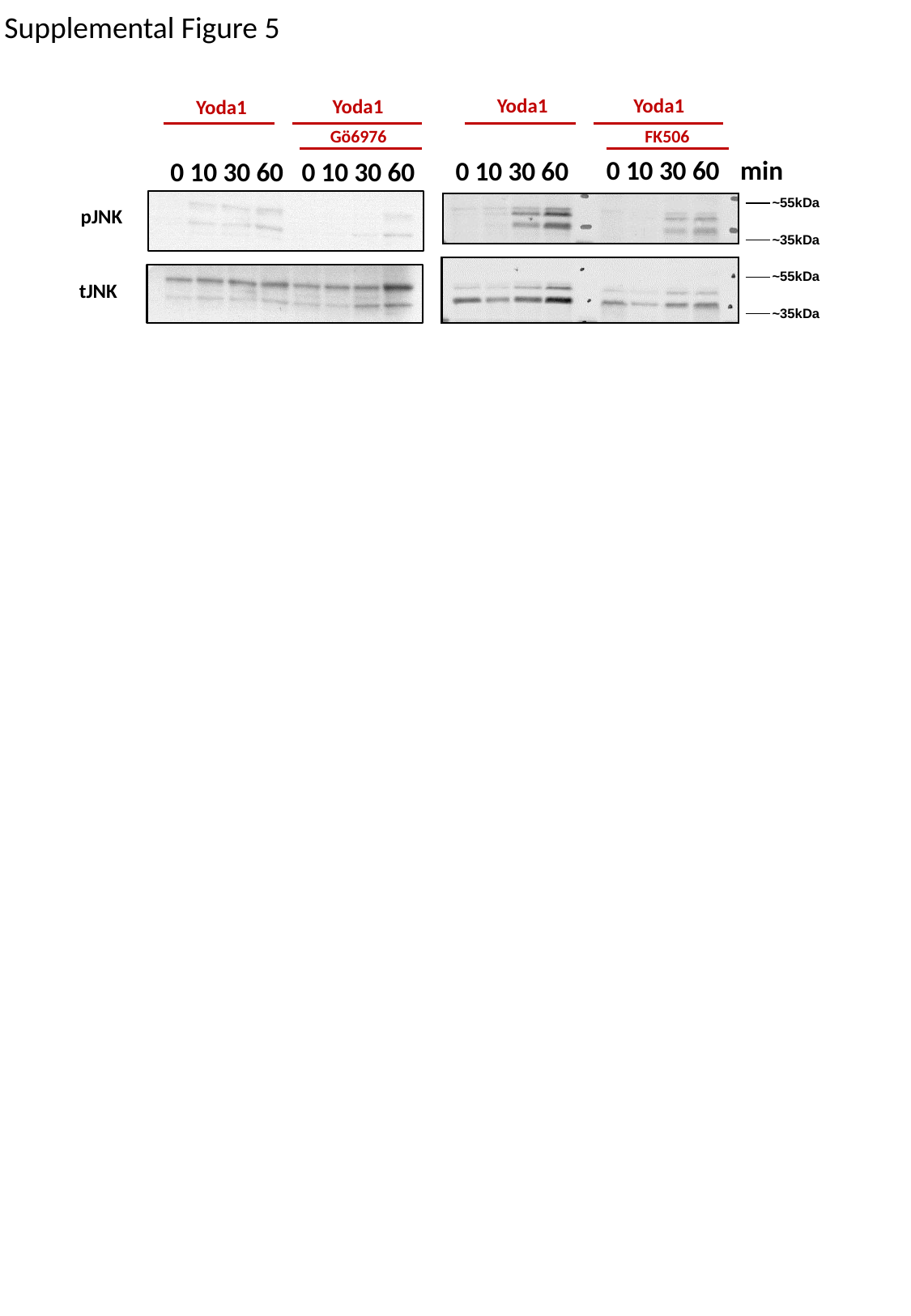

Supplemental Figure 5
Yoda1
Yoda1
Yoda1
Yoda1
Gö6976
FK506
min
0 10 30 60
0 10 30 60
0 10 30 60
0 10 30 60
~55kDa
pJNK
~35kDa
~55kDa
tJNK
~35kDa

## Slide 6
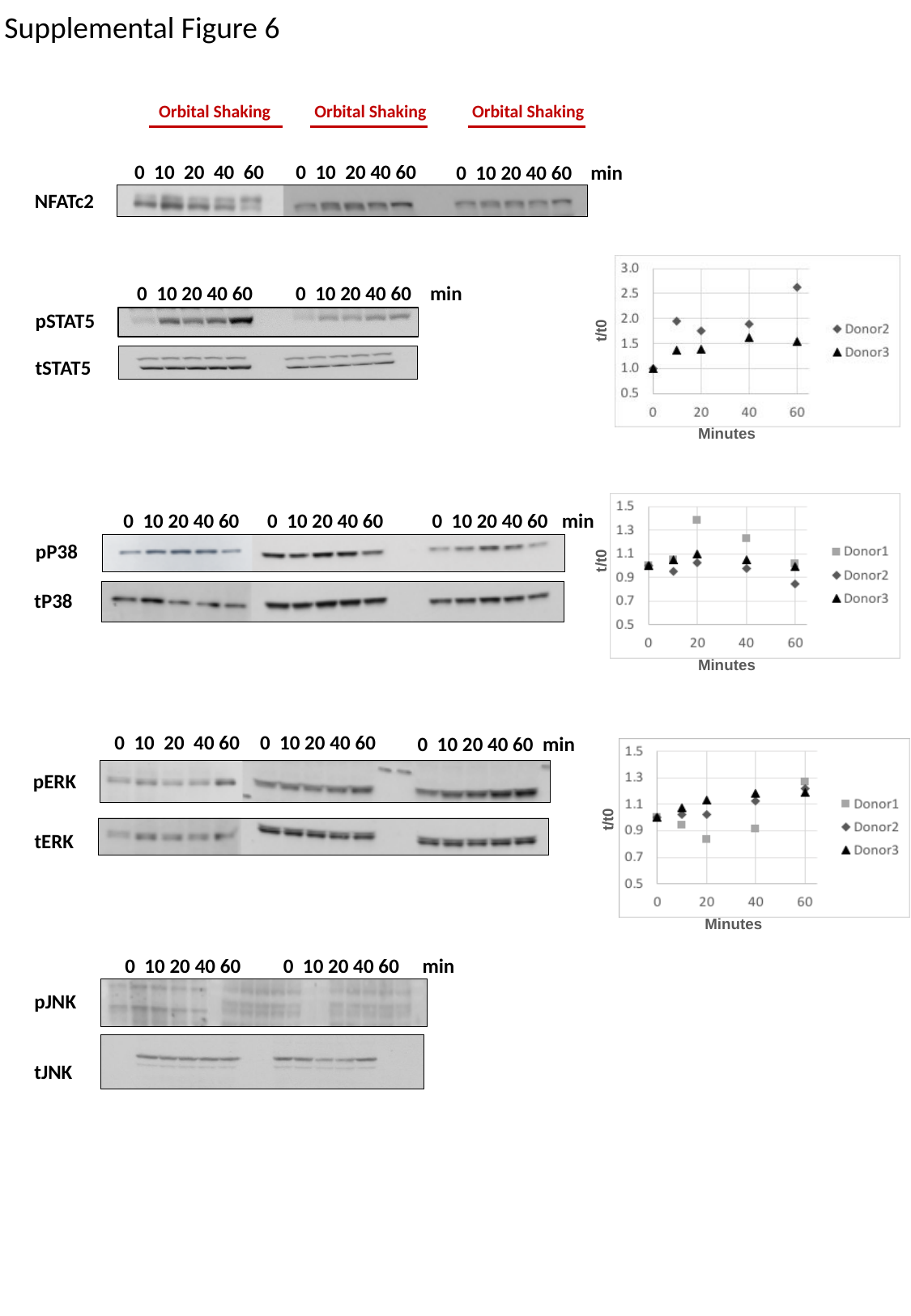

Supplemental Figure 6
Orbital Shaking
Orbital Shaking
Orbital Shaking
0 10 20 40 60
0 10 20 40 60
0 10 20 40 60 min
 NFATc2
0 10 20 40 60
0 10 20 40 60 min
pSTAT5
t/t0
tSTAT5
Minutes
0 10 20 40 60
0 10 20 40 60
0 10 20 40 60 min
pP38
t/t0
tP38
Minutes
0 10 20 40 60
0 10 20 40 60
0 10 20 40 60 min
pERK
t/t0
tERK
Minutes
0 10 20 40 60 min
0 10 20 40 60
pJNK
tJNK

## Slide 7
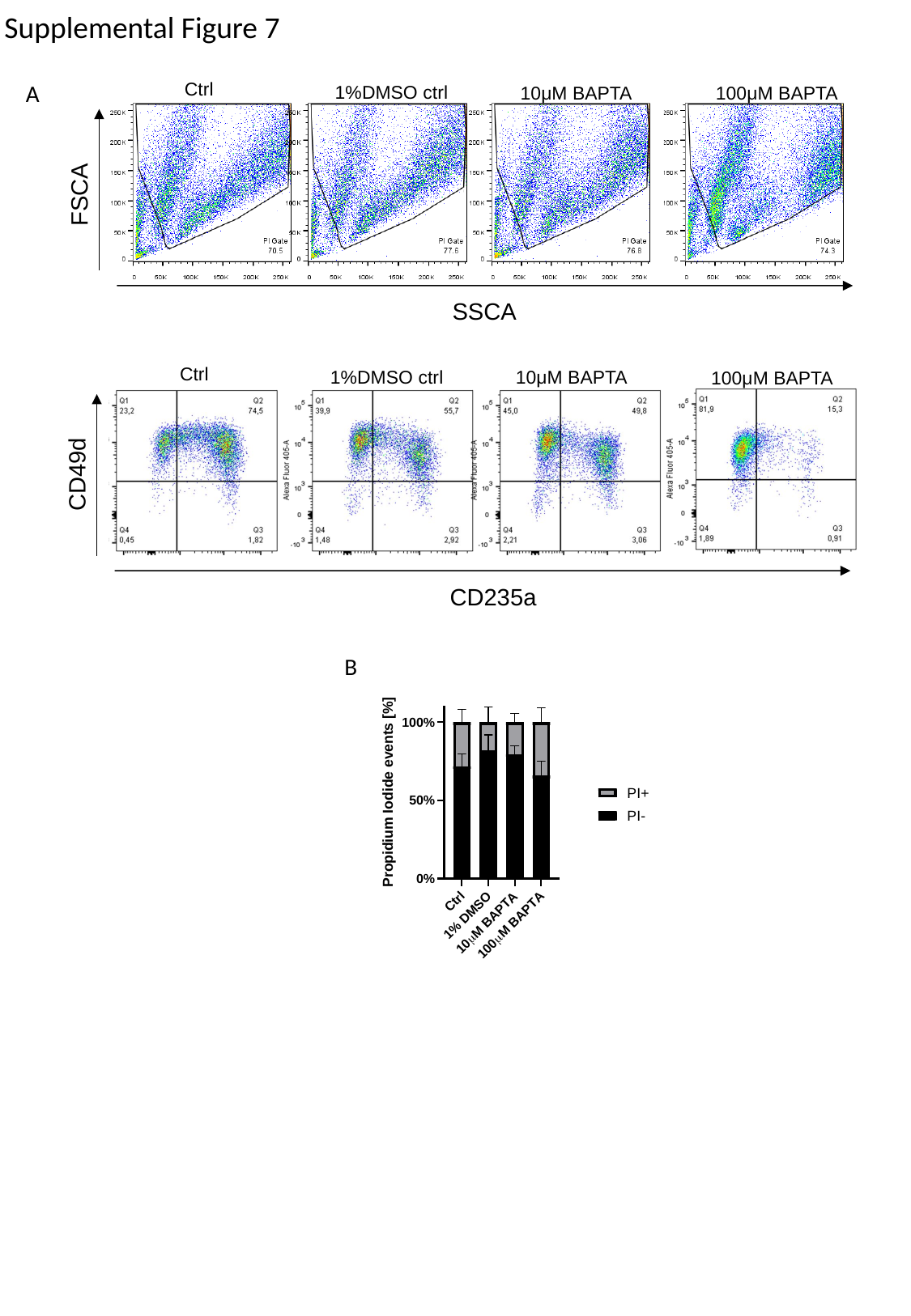

Supplemental Figure 7
A
Ctrl
1%DMSO ctrl
10μM BAPTA
100μM BAPTA
FSCA
SSCA
Ctrl
1%DMSO ctrl
10μM BAPTA
100μM BAPTA
CD49d
CD235a
B

## Slide 8
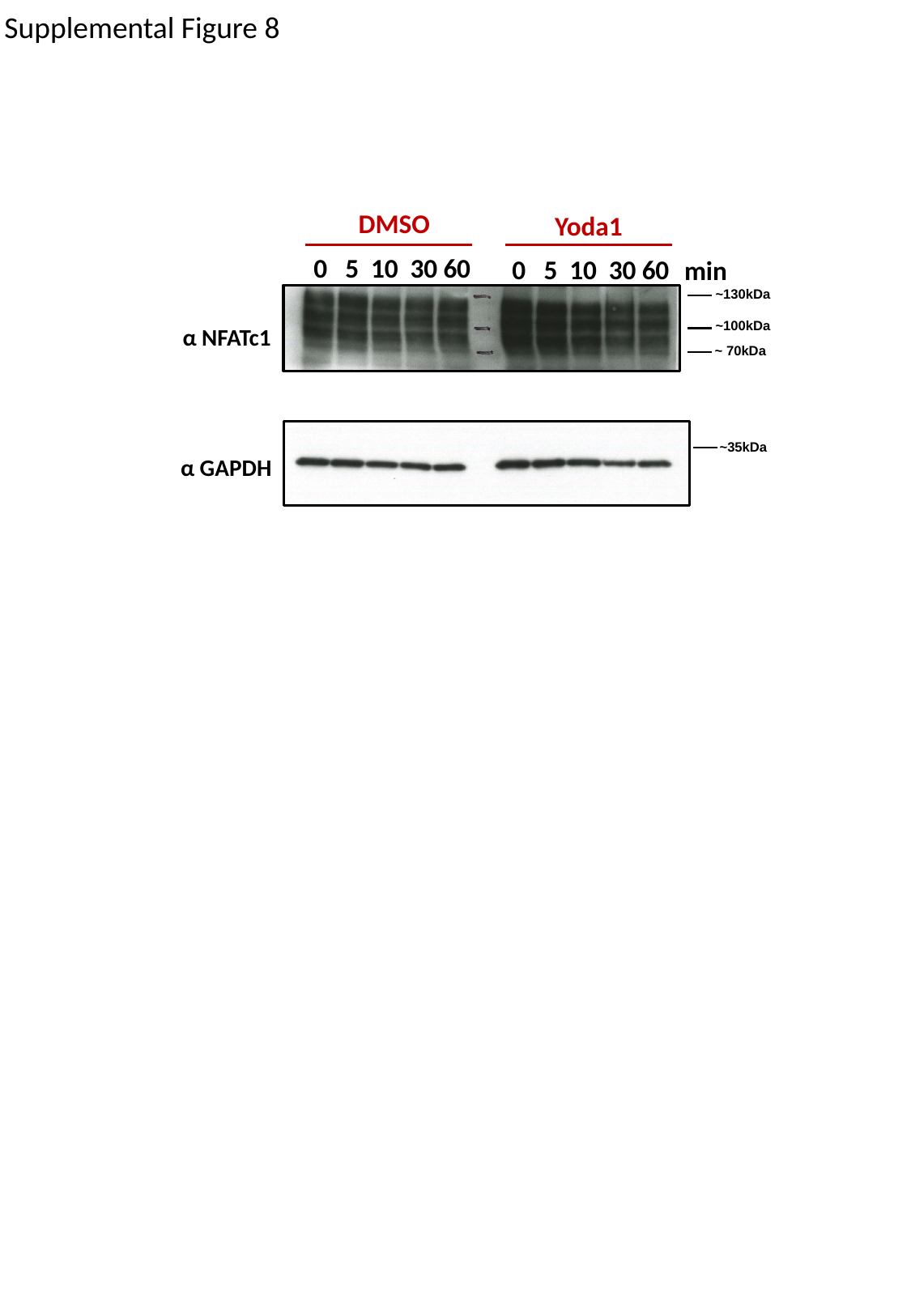

Supplemental Figure 8
DMSO
Yoda1
0 5 10 30 60
0 5 10 30 60
min
~130kDa
~100kDa
α NFATc1
~ 70kDa
~35kDa
α GAPDH
